# On determining conditions and suitable locations for fish survival by using the solution of the two coupled pollution and aeration equations

**DOI:** 10.1038/s41598-023-33368-9

**Published:** 2023-04-21

**Authors:** Philopatir B. Raafat, Fayez N. Ibrahim, Ahmed Saleh

**Affiliations:** 1 Department of Mathematics, Faculty of Science, Ain Shams University, Cairo, Egypt; 2 Department of Basic Science, Egyptian Academy for Engineering and Advanced Technology Affiliated to The Ministry of Military Production, Cairo, Egypt

**Keywords:** Hydrology, Mathematics and computing

## Abstract

The coupled equations of pollution and aeration for flow in a river were studied under generalized assumptions in terms of parameter dependency on space and time, as well as general boundary constraints. An analytical solution was obtained in the steady-state case. Also, the system was solved in its unsteady state numerically in a dimensionless form using the finite difference scheme. The effect of different parameters controlling the flow (such as the velocity, Peclet number, injected pollutants, and so on…) was studied. Investigations indicate that the special cases of the proposed model (i.e., uniform distribution of pollutant and Dissolved Oxygen concentrations, and zero injected pollutants along the river) give results that agree with the previous studies. This simple model helps in understanding the behavior of the pollution-aeration process and its relation to the injected pollution along a river and its effect on fish survival. A simple procedure was discussed in this study to help in regulating farming, industrial, and urban practices and impose restrictions if necessary. This study determines with accuracy the intervals of the river at which fish can survive at a given time, as well as the maximum amount of pollutants allowed to be injected along the river for fish survival.

## Introduction

Water pollution is one of the most important problems that directly affect human lives, fish survival, and environmental health in general. Hence, various attempts are made to search for affordable, efficient, and environmentally friendly water systems. Firstly, solar energy-based technologies^1^ are proposed by researchers^2^ as a cost-effective energy source in solar water pumps. Secondly, methods for heavy metal removal are being discussed^3^. Thirdly, biological wastes that can be treated by dissolved oxygen (DO) are modeled^4^, which is the focus of the current study. Predicting pollutant concentration in advance will reduce the negative effects drastically^5^. Tons of fish die every year as a consequence of water pollution^6^. Thus, mathematical models describing solute transport existed as early as the 1920s. Generally, the two main solute transport models are those based on the advection–diffusion equation (ADE), with different assumptions concerning initial and boundary conditions, the dependence of the parameters on space and time, and their generalizations in two and three dimensions. The other is the aeration model, which consists of two coupled non-linear partial differential equations (PDEs) with their respective conditions. The coupling occurs because the oxygen reacts with the pollutants that result from industrial and agricultural areas, producing harmless compounds. The aeration model is a powerful tool to help impose restrictions on urban and farming practices. The importance of this model is in enabling simulation scenarios to be tested for fish survival, which usually requires that the DO concentration be taken to be above 30% of the saturated DO concentration^7^. The pollution model has many analytical solutions for different cases. For instance, Shukla^8^ discussed the unsteady transport dispersion of nonconservative pollutants with time-dependent periodic waste discharge concentration. Jaiswal et al.^9^ obtained analytical solutions for the temporally and spatially dependent solute dispersion of pulse-type input concentration in one-dimensional semi-infinite media. Manitcharoen and Pimpunchat^10^ acquired analytical and numerical solutions of pollution concentration with uniformly and exponentially increasing forms of sources. Yadav and Kumar^11^ solved the space and time-dependent ADE and obtained an analytical solution of two‑dimensional conservative solute transport in a heterogeneous porous medium for varying input point sources. Saleh et al.^12^ solved the one-dimensional ADE describing exponential variations in pollutant concentration numerically and analytically and captured results regarding the remediation of pollution by releasing clean water. However, the aeration model is extremely difficult to have exact analytical solutions due to the non-linearity of the PDEs, which leads to two ways. Either to assume linearity and get analytical solutions or to use numerical methods in the non-linear case. Pimpunchat et al.^4^ modelled river pollution and its removal by aeration and obtained steady-state analytical solutions for some special cases. Ibrahim et al.^6^ obtained an analytical solution for the unsteady state aeration model in its linear case using the Laplace transformation technique. Hadhouda and Hassan^13^ obtained a numerical solution for the non-linear aeration model for two case studies. Studies on modelling the connection between survival of fish and fish population dynamics have been carried out^14–19^. On the experimental side, the study of aeration systems is a rich and active research area. Different aeration technologies and their importance are discussed by scientists with a view on design^20–23^.

This study aims to consider different solute transport models, such as time and space-dependent models, sinusoidally varying and uniform input sources, and initially decreasing pollutant concentrations along the river. Then use the explicit finite difference method to obtain a numerical solution of the proposed aeration model in its dimensionless form. In the steady-state case, an analytical solution is obtained and used to verify the validity of the numerical solution. The impact of different parameters is studied to validate the proposed aeration model and its compliance with the previous studies, thus generalizing them. Furthermore, we discuss a simple procedure to calculate the maximum pollutants allowed to be injected along the river to guarantee fish survival. The same procedure works for determining the best interval along the river and the time for placing fish farms. Also, such data is the basis for imposing any kind of regulations and restrictions on industrial, farming, and urban practices. Further studies using unconditionally-stable numerical methods would allow for wider parameter values and thus more flexibility in the real-life application process. The novel contribution of this study lies in generalizing the aeration model by considering space and time-dependent parameters as well as implementing generalized boundary conditions. Furthermore, we discussed how to use this simple model to find the injected pollutant concentration threshold for fish survival at a given time and the best place along the river to place fish farms.

## The governing equations

The coupled non-linear PDEs modelling pollutant and DO concentrations in a river can be written as^4,10,11^:

1 $$R\frac{{\partial \left( {AP} \right)}}{\partial t} = \frac{\partial }{\partial x}\left( {D_{P} \frac{{\partial \left( {AP} \right)}}{\partial x} - uAP} \right) - K_{P} \frac{X}{X + k}AP + q\left( {1 - \exp \left( { - \lambda x} \right)} \right)$$

2 $$R\frac{{\partial \left( {AX} \right)}}{\partial t} = \frac{\partial }{\partial x}\left( {D_{X} \frac{{\partial \left( {AX} \right)}}{\partial x} - uAX} \right) - K_{X} \frac{X}{X + k}AP + \alpha \left( {S - X} \right)$$

where $R$ is the retardation factor, $A({\text{km}}^{2} )$ is the cross-section area of the river which is taken to be constant, $P(x,t)\;({\text{kg}}\,{\text{km}}^{ - 3} )$ is the pollutant concentration, $X(x,t)\,({\text{kg}}\,{\text{km}}^{ - 3} )$ is the DO concentration, $D_{p} (x,t)\,({\text{km}}^{2} \,{\text{day}}^{ - 1} )$ is the diffusion coefficient of the pollutant concentration, $D_{X} (x,t)\,({\text{km}}^{2} \,{\text{day}}^{ - 1} )$ is the diffusion coefficient of DO, $u(x,t)\,({\text{km}}\,{\text{day}}^{ - 1} )$ is the river’s velocity, $K_{P} (x,t) ({\text{day}}^{ - 1} )$ is the degradation rate coefficient for pollution at a constant temperature, $K_{X} (x,t) ({\text{day}}^{ - 1} )$ is the degradation rate coefficient for DO at a constant temperature, $k({\text{kg}}\,{\text{km}}^{ - 3} )$ is the half-saturated oxygen demand concentration for pollution decay, $q(x,t)\,({\text{kg}}\,{\text{km}}^{ - 1} \,{\text{day}}^{ - 1} )$ is the rate of the injected pollutant discharge, $\lambda ({\text{km}}^{ - 1} )$ is an arbitrary constant of pollution source terms, $\alpha (x,t)\,({\text{km}}^{2} {\text{day}}^{ - 1} )$ is the mass transfer of oxygen from air to water and $S({\text{kg}}\,{\text{km}}^{ - 3} )$ is the saturated oxygen concentration.

The flow domain parameters are considered to be spatially and temporally dependent so we have:

3 $$\begin{aligned} & R = R_{0} {\text{f}}\left( {\text{x}} \right){ };D_{P} = D_{{P_{0} }} F\left( {x,t} \right);\;D_{X} = D_{{X_{0} }} F\left( {x,t} \right);\;u = u_{0} F\left( {x,t} \right);\;q = q_{0} F\left( {x,t} \right) \\ & K_{P} = K_{1} F\left( {x,t} \right);\;K_{X} = K_{2} F\left( {x,t} \right);\;\alpha = \alpha_{0} F\left( {x,t} \right) \\ & {\text{such}}\;{\text{that}}\;F(x,t) = g(mt)\;{\text{f}}\left( {\text{x}} \right)\;{\text{and}}\;{\text{f}}\left( {\text{x}} \right) = {\text{exp}}( - ({\text{ax}} + {\text{b}})), \\ \end{aligned}$$

where a $({\mathrm{km}}^{-1})$ is a heterogeneity parameter and b is an arbitrary dimensionless constant^11^, ${R}_{0}$ is the initial retardation factor, ${D}_{{P}_{0}}$ is the initial pollution diffusion coefficient, ${D}_{{X}_{0}}$ is the initial DO diffusion coefficient, ${u}_{0}$ is the initial velocity component, ${q}_{0}$ is the initial rate of the injected pollutant discharge, ${K}_{1}$ is the initial degradation rate coefficient for pollution at a constant temperature, ${K}_{2}$ is the initial degradation rate coefficient for DO at a constant temperature, ${\alpha }_{0}$ is the initial oxygen mass transfer, $m$ (day^-1^) is a constant. We choose $g\left(mt\right)$ such that for $t=0$ or $m=0$ we have $g\left(mt\right)=1$ which ensures that the nature of the initial condition doesn’t change in the new time domain $T$ (day) which is defined by^24^:

4 $$T={\int }_{0}^{t}g(mt)dt$$

Equations (3 and 4) transform Eqs. (1 and 2) into:

5 $$R_{0} \frac{\partial P}{{\partial T}} = D_{{P_{0} }} \frac{{\partial^{2} P}}{{\partial x^{2} }} - U_{P} \frac{\partial P}{{\partial x}} + \left\{ {au_{0} - K_{1} \frac{X}{X + k}} \right\}{ }P + q_{1} \left( {1 - \exp \left( { - \lambda x} \right)} \right)$$

6 $${R}_{0}\frac{\partial X}{\partial T}={D}_{{X}_{0}}\frac{{\partial }^{2}X}{\partial {x}^{2}}-{U}_{X}\frac{\partial X}{\partial x}+{au}_{0}X-{K}_{2}\frac{X}{X+k}P+{\alpha }_{1}(S-X)$$

where $${U}_{P}=\left\{{u}_{0}+a{D}_{{p}_{0}}\right\}$$, $${U}_{X}=\left\{{u}_{0}+{aD}_{{X}_{0}}\right\}$$, $${q}_{1}=\frac{{q}_{0}}{A}$$; $${\alpha }_{1}=\frac{{\alpha }_{0}}{A}$$.

Assuming the initial solute concentration to be an exponentially decreasing function of the space variable^9,13^, a diurnal flow variation from a wastewater treatment plant with a constant discharge is modelled approximately by a sinusoidal wave^8,25^. Also, far from the origin ($x=0$), it is assumed that there is no concentration exchange with the system. Hence the initial and boundary conditions are:

7 $$P\left( {x,t} \right) = P_{0} + P_{1} \exp \left[ { - \eta_{1} x} \right],\quad x \ge 0,\quad t = 0$$

8 $$P\left( {x,t} \right) = P_{2} + P_{3} \sin \left( {\omega t} \right),\quad x = 0,\quad t > 0$$

9 $$\frac{\partial P}{{\partial x}} = 0,\quad x \to \infty ,\quad t \ge 0$$

10 $$X\left( {x,t} \right) = X_{0} + X_{1} \exp \left[ { - \eta_{2} x} \right],\quad x \ge 0,\quad t = 0$$

11 $$X\left( {x,t} \right) = X_{2} + X_{3} \cos \left( {\omega t} \right),\quad x = 0,\quad t > 0$$

12 $$\frac{\partial X}{{\partial x}} = 0,\quad x \to \infty ,\quad t \ge 0$$

where ${P}_{0}$ and ${X}_{0}$ are the initial uniform concentrations for pollution and DO as $x\to \infty$,${P}_{1}$ and ${X}_{1}$ are the uniform pollution and DO concentrations respectively when ${\eta }_{1}={ \eta }_{2}={P}_{0}={X}_{0}=0$, ${P}_{2}$ and ${X}_{2}$ are the average added pollution and DO concentrations respectively, ${P}_{3}$ and ${X}_{3}$ are the amplitudes of pollution and DO variation respectively, ${\eta }_{1}$ and ${\eta }_{2}$ are the initial pollution and DO decay lengths respectively, $\omega$ is the frequency of the concentration variations^8^. It is known that the behavior of variation of the pollutant concentration is opposite to that of the DO. Hence, it is convenient to choose the sine function in Eq. (8) and the cosine function in Eq. (11).

It is generally more convenient to write equations in dimensionless variables. Let ${\gamma }_{1}=a{u}_{0}$.

Take ${\gamma }_{1}$ as the time scale, ${u}_{0}$ as the velocity scale, ${P}_{2}$ and ${X}_{2}$ as the concentration scales and use the symbol (*) to denote a dimensionless quantity, hence:

13 $$\begin{aligned} & P^{*} = \frac{P}{{P_{2} }};P_{1}^{*} = \frac{{P_{1} }}{{P_{2} }};P_{2}^{*} = 1;X^{*} = \frac{X}{{X_{2} }};X_{1}^{*} = \frac{{X_{1} }}{{X_{2} }};X_{2}^{*} = 1;U_{P}^{*} = \frac{{U_{P} }}{{u_{0} }};U_{X}^{*} = \frac{{U_{X} }}{{u_{0} }};z^{*} = \frac{{ \gamma_{1} x}}{{u_{0} }};T^{*} = \gamma_{1} T ; \\ & \mu^{*} = \frac{{q_{1} }}{{P_{2} \gamma_{1} }};\lambda^{*} = \frac{{u_{0} \lambda }}{{\gamma_{1} }};K_{2}^{*} = \frac{{K_{2} }}{{\gamma_{1} }};K_{1}^{*} = \frac{{K_{1} }}{{\gamma_{1} }};k^{*} = \frac{k}{{P_{2} }};S^{*} = \frac{S}{{X_{2} }};\gamma_{1}^{*} = 1;\alpha_{1}^{*} = \frac{{\alpha_{1} }}{{\gamma_{1} }}; \eta_{1}^{*} = \frac{{u_{0} \eta_{1} }}{{\gamma_{1} }} \\ & \eta_{2}^{*} = \frac{{u_{0} \eta_{2} }}{{\gamma_{1} }};\omega^{*} = \frac{\omega }{{\gamma_{1} }};P_{0}^{*} = \frac{{P_{0} }}{{P_{2} }};P_{3}^{*} = \frac{{P_{3} }}{{P_{2} }};X_{0}^{*} = \frac{{X_{0} }}{{X_{2} }};X_{3}^{*} = \frac{{X_{3} }}{{X_{2} }}; \\ \end{aligned}$$

Equation (13) transforms Eqs. (5–12) into:

14 $$R_{0} \frac{{\partial P^{*} }}{{\partial T^{*} }} = \frac{1}{{Pe_{{P^{*} }} }}\frac{{\partial^{2} P^{*} }}{{\partial z^{*2} }} - U_{P}^{*} \frac{{\partial P^{*} }}{{\partial z^{*} }} + \left\{ {1 - K_{1}^{*} \frac{{X^{*} }}{{X^{*} + k^{*} }}} \right\} P^{*} +\upmu ^{*} \left( {1 - \exp \left( { - \lambda^{*} z^{*} } \right)} \right)$$

15 $$R_{0} \frac{{\partial X^{*} }}{{\partial T^{*} }} = \frac{1}{{Pe_{{X^{*} }} }}\frac{{\partial^{2} X^{*} }}{{\partial z^{*2} }} - U_{X}^{*} \frac{{\partial X^{*} }}{{\partial z^{*} }} + X^{*} - K_{2}^{*} \frac{{X^{*} }}{{X^{*} + k^{*} }}P^{*} + \alpha_{1}^{*} \left( {S^{*} - X^{*} } \right)$$

16 $$P^{*} \left( {z^{*} ,T^{*} } \right) = P_{0}^{*} + P_{1}^{*} {\text{exp}}\left[ { - \eta_{1}^{*} z^{*} } \right],\quad z^{*} \ge 0,\quad T^{*} = 0$$

17 $$P^{*} \left( {z^{*} ,T^{*} } \right) = 1 + P_{3}^{*} \sin \left( {\omega^{*} T^{*} } \right),\quad z^{*} = 0,\quad T^{*} > 0$$

18 $$\frac{{\partial P^{*} }}{{\partial z^{*} }} = 0,\quad z^{*} \to \infty \quad T^{*} \ge 0$$

19 $$X^{*} \left( {z^{*} ,T^{*} } \right) = X_{0}^{*} + X_{1}^{*} {\text{exp}}\left[ { - \eta_{2}^{*} z^{*} } \right],\quad z^{*} \ge 0,\quad T^{*} = 0$$

20 $$X^{*} \left( {z^{*} ,T^{*} } \right) = 1 + X_{3}^{*} \cos \left( {\omega^{*} T^{*} } \right),\quad z^{*} = 0,\quad T^{*} > 0$$

21 $$\frac{{\partial X^{*} }}{{\partial z^{*} }} = 0,\quad z^{*} \to \infty \quad T^{*} \ge 0$$

where $Pe_{{P^{*} }} = \frac{{u_{0}^{2} }}{{D_{{p_{0} }} \gamma_{1} }}$ and $Pe_{{X^{*} }} = \frac{{u_{0}^{2} }}{{D_{{X_{0} }} \gamma_{1} }}$ are the pollution and DO Peclet numbers respectively.

## Problem description

It is noticed that the term ${\upmu }^{*}(1-\mathrm{exp}\left(-{\lambda }^{*}{z}^{*}\right))$ in Eq. (14) and the term $\mathrm{exp}\left[-{{ \eta }_{1}}^{*}{z}^{*}\right]$ in Eq. (16) have opposite effects on the pollutant concentration so we only consider the two cases where only one of them is present in the model.

### Case study one (${P}_{1}^{*}={X}_{1}^{*}=0$)

In this case, it is assumed that pollutants are injected along the river at a rate ${\upmu }^{*}$ and that its value in the upstream is lower than its value in the downstream which is modelled as ${\upmu }^{*}(1-\mathrm{exp}\left(-{\lambda }^{*}{z}^{*}\right))$^10^.

### Case study two (${\lambda }^{*}=0$ or ${\upmu }^{*}=0$)

In this case, it is assumed that no pollutants are injected along the river and that the pollutant and DO concentrations are initially an exponentially decreasing function of the space variable (Eqs. 16 and 19)^9^.

## Analytical solution for the steady state case

It is well known that analytical solutions are important in validating numerical solutions. However, the exact analytical solution of Eqs. (14 and 21) is very difficult if not impossible especially for the nonlinear case. Also, previous studies indicates that solution of the unsteady case is very tedious^4,6^. To simplify the equations, we will consider the steady state and ${k}^{*}=0$, ${Pe}_{{P}^{*}}={Pe}_{{X}^{*}}=Pe$ and ${{U}_{P}}^{*}={{U}_{X}}^{*}={U}^{*}$. Hence, Eqs. (14–21) take the form:

22 $$\frac{1}{Pe}\frac{{d^{2} P^{*} \left( {z^{*} } \right)}}{{dz^{*2} }} - U^{*} \frac{{dP^{*} \left( {z^{*} } \right)}}{{dz^{*} }} + \left\{ {1 - K_{1}^{*} } \right\}P^{*} \left( {z^{*} } \right) + \mu^{*} \left( {1 - \exp \left( { - \lambda^{*} z^{*} } \right)} \right) = 0$$

23 $$\frac{1}{Pe}\frac{{d^{2} X^{*} \left( {z^{*} } \right)}}{{dz^{*2} }} - U^{*} \frac{{dX^{*} \left( {z^{*} } \right)}}{{dz^{*} }} + X^{*} \left( {z^{*} } \right) - K_{2}^{*} P^{*} \left( {z^{*} } \right) + \alpha_{1}^{*} \left( {S^{*} - X^{*} } \right){ } = 0$$

24 $$P^{*} \left( {z^{*} } \right) = 1,\quad z^{*} = 0$$

25 $$\frac{{dP^{*} \left( {z^{*} } \right)}}{{dz^{*} }} = 0,\quad z^{*} \to \infty$$

26 $$X^{*} \left( {z^{*} } \right) = 1,\quad z^{*} = 0$$

27 $$\frac{{dX^{*} \left( {z^{*} } \right)}}{{dz^{*} }} = 0,\quad z^{*} \to \infty$$

The solution of Eqs. (22–27) may be written as:

28 $$P^{*} \left( {z^{*} } \right) = - \left[ {\frac{{f_{1} + C_{1} f_{2} }}{{\left( {K_{1}^{*} - 1} \right)\psi }}} \right]$$

where

$$f_{1} = (K_{1}^{*} - 1)Pe \mu^{*} \exp ( - \lambda^{*} z^{*} ) + \mu^{*} (Pe - K_{1}^{*} Pe + Pe U^{*} \lambda^{*} + \lambda^{*2} )$$

$$f_{2} = (K_{1}^{*} - 1)(Pe - K_{1}^{*} Pe + PeU^{*} \lambda ^{*} + \lambda ^{{*2}} )\exp \left. {\left( {\frac{1}{2}(PeU^{*} z^{*} - \sqrt {Pe} \sqrt { - 4 + 4K_{1}^{*} + PeU^{{*2}} } z^{*} } \right)} \right)$$

$$C_{1} = \frac{{ - ((Pe - K_{1}^{*} Pe + Pe U^{*} \lambda^{*} + \lambda^{*2} )(K_{1}^{*} - 1 - \mu^{*} )) + Pe \mu^{*} (K_{1}^{*} - 1)}}{{(K_{1}^{*} - 1)\psi }}$$; $$\psi = ( - \lambda^{*2} + Pe( - 1 + K_{1}^{*} - U^{*} \lambda^{*} ))$$.

29 $$X^{*} \left( {z^{*} } \right) = \frac{ - 64}{\beta }\quad \left( {f_{3} + f_{4} + f_{5} } \right)$$

where

$$\begin{aligned} & f_{3} = \mu^{*} K_{2}^{*} Pe^{2} (K_{1}^{*} - 1) (\alpha_{1}^{*} - 1)(\alpha_{1}^{*} - K_{1}^{*} )\exp ( - \lambda^{*} z^{*} ) + (K_{1}^{*} - \alpha_{1}^{*} ) \\ & \quad (Pe - \alpha_{1}^{*} Pe + Pe U^{*} \lambda^{*} + \lambda^{*2} )( - \lambda^{*2} + Pe(K_{1}^{*} - U^{*} \lambda^{*} - 1)) \\ & \quad (\alpha_{1}^{*} S^{*} (K_{1}^{*} - 1) - \mu^{*} K_{2}^{*} ) \\ \end{aligned}$$

$$\begin{aligned} f_{4} & = - \left[ {K_{2}^{*} \left( {\alpha _{1}^{*} - 1} \right)\left( { - \lambda ^{{*2}} + Pe(\alpha _{1}^{*} - U^{*} \lambda ^{*} - 1} \right)} \right) \\ & \quad \left. {\left. {\left( {\lambda ^{{*2}} \left( {\mu ^{*} + 1 - K_{1}^{*} } \right) + Pe\left( {1 + K_{1}^{{*2}} - K_{1}^{*} (2 + U^{*} \lambda ^{*} + U^{*} \lambda ^{*} (1 + \mu ^{*} } \right)} \right)} \right)} \right) \\ & \quad \left. {\exp \left( { - \frac{1}{2}\left( { - PeU^{*} + \sqrt {Pe} \sqrt { - 4 + 4K_{1}^{*} + PeU^{{*2}} } } \right)z^{*} } \right)} \right] \\ \end{aligned}$$

$$f_{5} = \frac{{C_{2} }}{64}\exp ( - \frac{1}{2}(Pe U^{*} + \sqrt {Pe} \sqrt { - 4 + 4\alpha_{1}^{*} + Pe U^{*2} } )z^{*} )$$

$${C}_{2}=-\beta +64\left({\mu }^{*}{K}_{2}^{*}{Pe}^{2}\left({K}_{1}^{*}-1\right) \left({{\alpha }_{1}}^{*}-1\right)\left({K}_{1}^{*}-{{\alpha }_{1}}^{*}\right)\right)+64\left({K}_{1}^{*}-{{\alpha }_{1}}^{*}\right)\left(Pe-{K}_{1}^{*} Pe+Pe {U}^{*} {\lambda }^{*}+{{\lambda }^{*}}^{2}\right)\left(Pe-{{\alpha }_{1}}^{*}Pe +Pe {U}^{*} {\lambda }^{*}+{{\lambda }^{*}}^{2}\right)\left({{\alpha }_{1}}^{*}{S}^{*}\left({K}_{1}^{*}-1\right)-{\mu }^{*}{K}_{2}^{*}\right)+64 {K}_{2}^{*} \left(1-{{\alpha }_{1}}^{*}\right)\left(Pe-{{\alpha }_{1}}^{*}Pe +Pe {U}^{*} {\lambda }^{*}+{{\lambda }^{*}}^{2}\right) [\left({{\lambda }^{*}}^{2}\left({\mu }^{*}+1-{K}_{1}^{*}\right)\right)+Pe\left(1+{{K}_{1}^{*}}^{2}-{K}_{1}^{*}\left(2+{U}^{*} {\lambda }^{*}+{U}^{*} {\lambda }^{*}\left(1+{\mu }^{*}\right)\right)\right)]$$

$$\beta = ((K_{1}^{*} - 1)$$ ($$4\alpha_{1}^{*} + Pe U^{*2} - Pe U^{*} - 4$$) ($$4\alpha_{1}^{*} - 4K_{1}^{*}$$) ($$Pe( - 4 + 4\alpha_{1}^{*} + Pe U^{*2} ) - (Pe U^{*} + 2\lambda^{*} )^{2} )$$$$(Pe - K_{1}^{*} Pe + Pe U^{*} \lambda^{*} + \lambda^{*2} )$$.

We have checked that Eqs. (28 and 29) satisfy Eqs. (22–27).

## Numerical solution

Analytical solutions of ADE with limited initial and boundary conditions have very few applications and are very lengthy^26^. The added difficulty of the non-linearity makes obtaining exact analytical solutions extremely hard, if not impossible. Numerical methods do not have such limitations, especially for arbitrary conditions. Thus, numerical solutions are obtained using a two-level explicit finite difference scheme. The truncation error is $O(\Delta {T}^{*},{(\Delta {z}^{*})}^{2})$ and can be reduced until the accuracy achieved is within error tolerance by taking suitable values of $\Delta {T}^{*}$ and $\Delta {z}^{*}$^27^. Hence, step-sizes Δ${z}^{*}$=1 and Δ ${T}^{*}$=0.002 along the ${z}^{*}$-domain and ${T}^{*}$-domain, are chosen respectively. The explicit finite difference method is employed to solve Eqs. (14–15) with initial and boundary conditions (16–21). The central difference scheme was used for $\frac{{\partial }^{2}{P}^{*}}{\partial {{z}^{*}}^{2}}$, $\frac{\partial {P}^{*}}{\partial {z}^{*}}$, $\frac{{\partial }^{2}{X}^{*}}{\partial {{z}^{*}}^{2}}$ and $\frac{\partial {P}^{*}}{\partial {z}^{*}}$ and a forward difference scheme for $\frac{\partial {P}^{*}}{\partial {T}^{*}}$ and $\frac{\partial {X}^{*}}{\partial {T}^{*}}$, with these substitutions, Eqs. (14–21) can be written as:

30 $$R_{0} P_{i,j + 1}^{*} = r_{1} P_{i - 1,j}^{*} + r_{2} P_{i,j}^{*} + r_{3} P_{i + 1,j}^{*} + \Delta T_{j}^{*} \left\{ {1 - K_{1}^{*} \frac{{X_{i,j}^{*} }}{{X_{i,j}^{*} + k^{*} }}} \right\}{ }P_{i,j}^{*} + \Delta T_{j}^{*} {\upmu }^{*} \left( {1 - \exp \left( { - \lambda^{*} z_{i}^{*} } \right)} \right)$$

31 $$R_{0} X_{i,j + 1}^{*} = r_{4} X_{i - 1,j}^{*} + r_{5} X_{i,j}^{*} + r_{6} X_{i + 1,j}^{*} - \Delta T_{j}^{*} K_{2}^{*} \frac{{X_{i,j}^{*} }}{{X_{i,j}^{*} + k^{*} }}P_{i,j}^{*} + \Delta T_{j}^{*} \alpha_{1}^{*} \left( {S^{*} - X_{i,j}^{*} } \right)$$

32 $$P_{i,0}^{*} = P_{0}^{*} + P_{1}^{*} {\text{exp}}\left[ { - \eta_{1}^{*} z_{i}^{*} } \right]$$

33 $$P_{0,j}^{*} = 1 + P_{3}^{*} \sin \left( {\omega^{*} T_{j}^{*} } \right)$$

34 $$P_{N,j}^{*} = P_{N - 1,j}^{*}$$

35 $$X_{i,0}^{*} = X_{0}^{*} + X_{1}^{*} {\text{exp}}\left[ { - \eta_{2}^{*} z_{i}^{*} } \right]$$

36 $$X_{i,0}^{*} = 1 + X_{3}^{*} \cos \left( {\omega^{*} T_{j}^{*} } \right)$$

37 $$X_{N,j}^{*} = X_{N - 1,j}^{*}$$

where *i* and *j* refer to the discrete step lengths Δ${z}^{*}$ and Δ${T}^{*}$ for the coordinates ${z}^{*}$ and ${T}^{*}$ respectively, and ${r}_{1}=\frac{\Delta {T}^{*} }{{Pe}_{{P}^{*}} (\Delta {{z}^{*})}^{2}}+\frac{{{U}_{P}}^{*}\Delta {T}^{*} }{2\Delta {z}^{*}}$; ${r}_{2}={R}_{0}-\frac{2 \Delta {T}^{*} }{{Pe}_{{P}^{*}} (\Delta {{z}^{*})}^{2}}$; ${r}_{3}=\frac{\Delta {T}^{*} }{{Pe}_{{P}^{*}} (\Delta {{z}^{*})}^{2}}-\frac{{{U}_{P}}^{*}\Delta {T}^{*} }{2\Delta {z}^{*}}$; ${r}_{4}=\frac{\Delta {T}^{*} }{{Pe}_{{X}^{*}} (\Delta {{z}^{*})}^{2}}+\frac{{{U}_{X}}^{*}\Delta {T}^{*} }{2\Delta {z}^{*}}$; ${r}_{5}={\Delta {T}^{*}+R}_{0}-\frac{2 \Delta {T}^{*} }{{Pe}_{{X}^{*}} (\Delta {{z}^{*})}^{2}}$; ${r}_{6}=\frac{\Delta {T}^{*} }{{Pe}_{{X}^{*}} (\Delta {{z}^{*})}^{2}}-\frac{{{U}_{X}}^{*}\Delta {T}^{*} }{2\Delta {z}^{*}}$; $N=\frac{{z}_{\infty }^{*} }{{ \Delta z}^{*}}$ is the grid dimension in the ${z}^{*}$ direction and ${z}_{\infty }^{*}$ is the distance measured from the origin at which $\frac{\partial {P}^{*}}{\partial {z}^{*}}\to 0$ and $\frac{\partial {X}^{*}}{\partial {z}^{*}}\to 0$.

Equations (30 and 31) represents a formula for ${P}_{i,j+1}^{*}$ and ${X}_{i,j+1}^{*}$ at the ${(\mathrm{i},\mathrm{j}+1)}^{th}$ mesh point in terms of known values along the jth time row.

## Results and discussions

**Figure 1:**
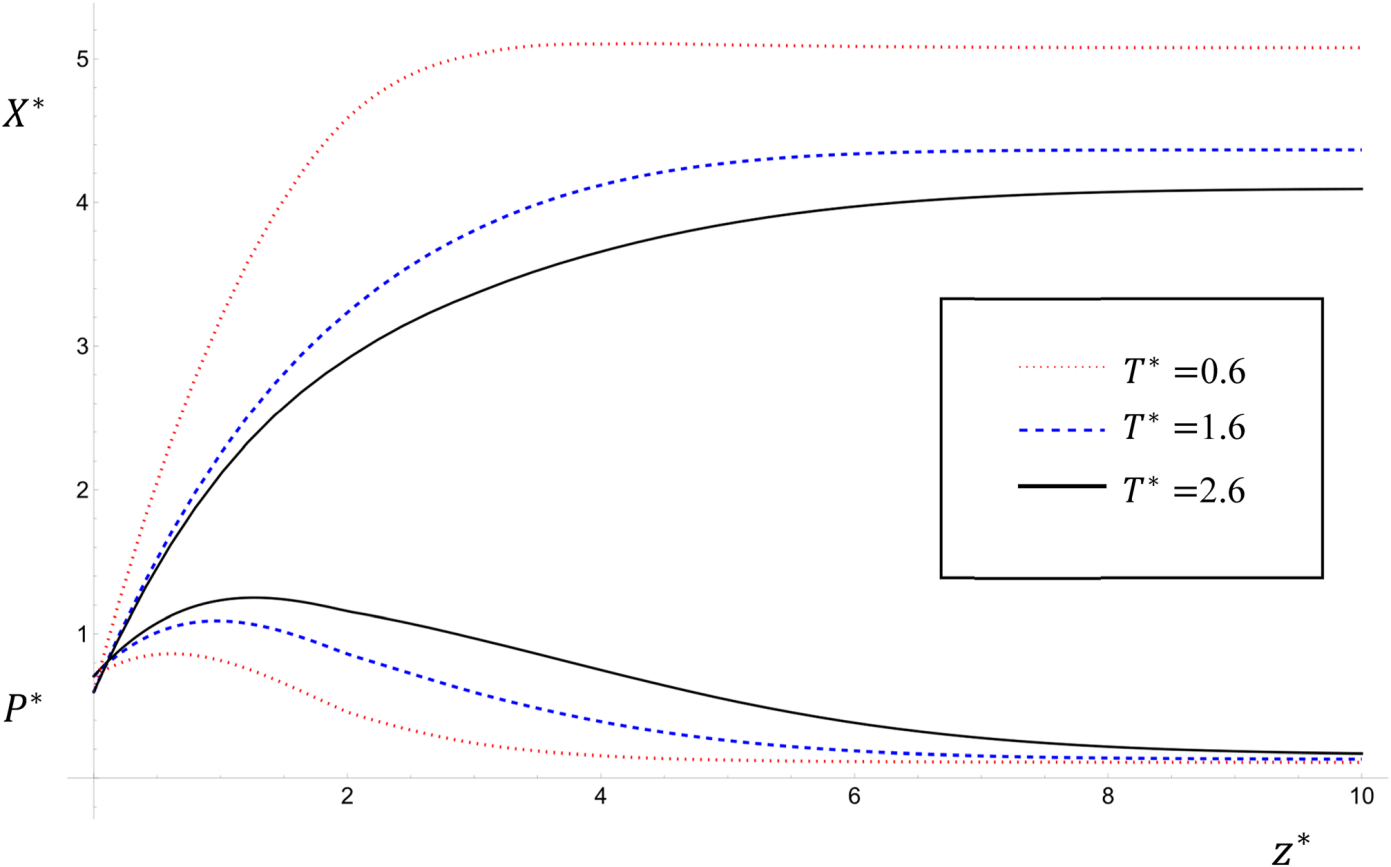
The variations of ${P}^{*}$ and ${X}^{*}$ with time ${T}^{*}$ for the values ${T}^{*}=0.6, 1.6, 2.6$ and ${U}^{*}={Pe}_{{P}^{*}}={Pe}_{{X}^{*}}={k}^{*}=1$; ${\upmu }^{*}=0$ for case study two.

**Figure 2:**
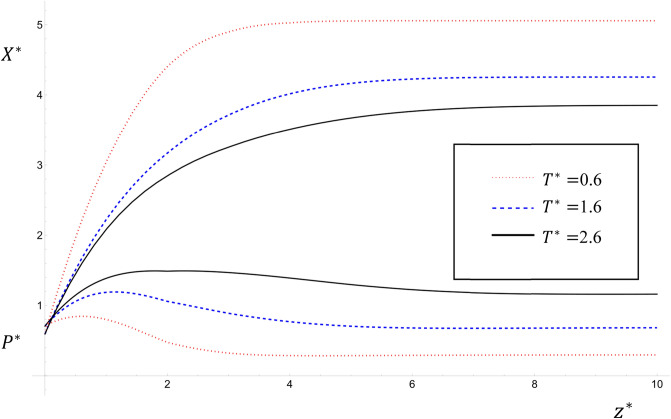
The variations of ${P}^{*}$ and ${X}^{*}$ with time ${T}^{*}$ for the values ${T}^{*}=0.6, 1.6, 2.6$ and ${U}^{*}={Pe}_{{P}^{*}}={Pe}_{{X}^{*}}={k}^{*}=1$; ${\upmu }^{*}=0.3$ for case study one.

**Figure 3:**
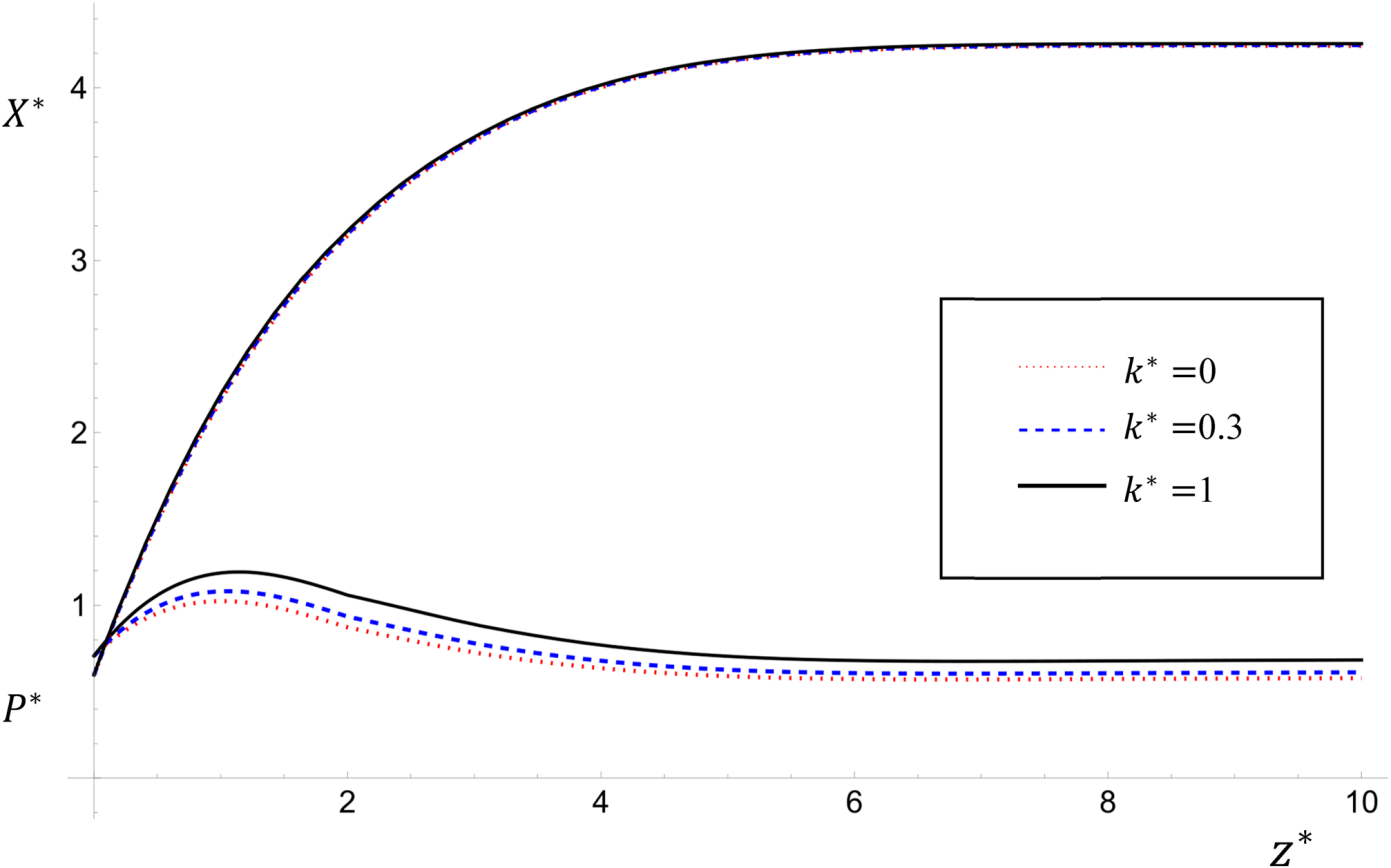
The variations of ${P}^{*}$ and ${X}^{*}$ with ${k}^{*}$ for the values ${k}^{*}=0, 0.3,1$ and ${T}^{*}=1.6$, ${Pe}_{{P}^{*}}={Pe}_{{X}^{*}}={U}^{*}=1$; ${\upmu }^{*}=0.3$ for case study one.

The numerical solutions of Eqs. (30–37) are illustrated in Figs. 1, 2 and 3 for the common input data: ${K}_{1}^{*}=1$; ${K}_{2}^{*}=0.5$; ${S}^{*}=$ ${{\alpha }_{1}}^{*}=2$; ${{\eta }_{1}}^{*}={{ \eta }_{2}}^{*}=0.98$; ${\omega }^{*}=2\pi$; ${\lambda }^{*}=0.6$; ${R}_{0}=1$; ${P}_{0}^{*}=0.1$; ${{P}_{1}}^{*}=0.5$; ${P}_{3}^{*}=0.5$; ${X}_{0}^{*}=6$; ${{X}_{1}}^{*}=2$; ${X}_{3}^{*}=0.5$; $0\le {z}^{*}\le 70$.

Figures 1 and 2 show the variations of ${P}^{*}$ and ${X}^{*}$ with ${T}^{*}$ for ${T}^{*}=0.6, 1.6, 2.6$ for case studies two and one respectively for the values ${U}^{*}={Pe}_{{P}^{*}}={Pe}_{{X}^{*}}={k}^{*}=1$. It is clear that as ${T}^{*}$ increases the value of ${P}^{*}$ increases and the value of ${X}^{*}$ decreases. This result agrees with that obtained by^6,13^. These figures emphasize the fact that the effect of the term ${\upmu }^{*}(1-\mathrm{exp}\left(-{\lambda }^{*}{z}^{*}\right))$ is apparent in making the corresponding values of ${P}^{*}$ in Fig. 2 bigger than in Fig. 1 especially far from the origin^10^. One application of this model is that it predicts the pollutant concentration in the future, which is essential in predicting the following two scenarios: (a) To determine with accuracy the intervals along the river with the highest pollution to focus on its remediation by effective methods at the suitable time. (b) To determine with accuracy the intervals along the river with the lowest pollution from which to withdraw clean water for different uses.

Since ${k}^{*}$ represents the half-saturated oxygen demand concentration for pollution decay, a direct consequence is its dominant effect on ${P}^{*}$ while its effect on ${X}^{*}$ is negligible. Figure 3 shows the variations of ${P}^{*}$ and ${X}^{*}$ with ${k}^{*}$ for ${k}^{*}=0, 0.3, 1$ for case study one for the values ${T}^{*}=1.6$ ; ${U}^{*}={Pe}_{{P}^{*}}={Pe}_{{X}^{*}}=1$. It is obvious that as ${k}^{*}$ increases, the value of ${P}^{*}$ also increases and the value of ${X}^{*}$ increases slightly but is almost unchanged. This result agrees with that obtained by^7,13^.

**Figure 4:**
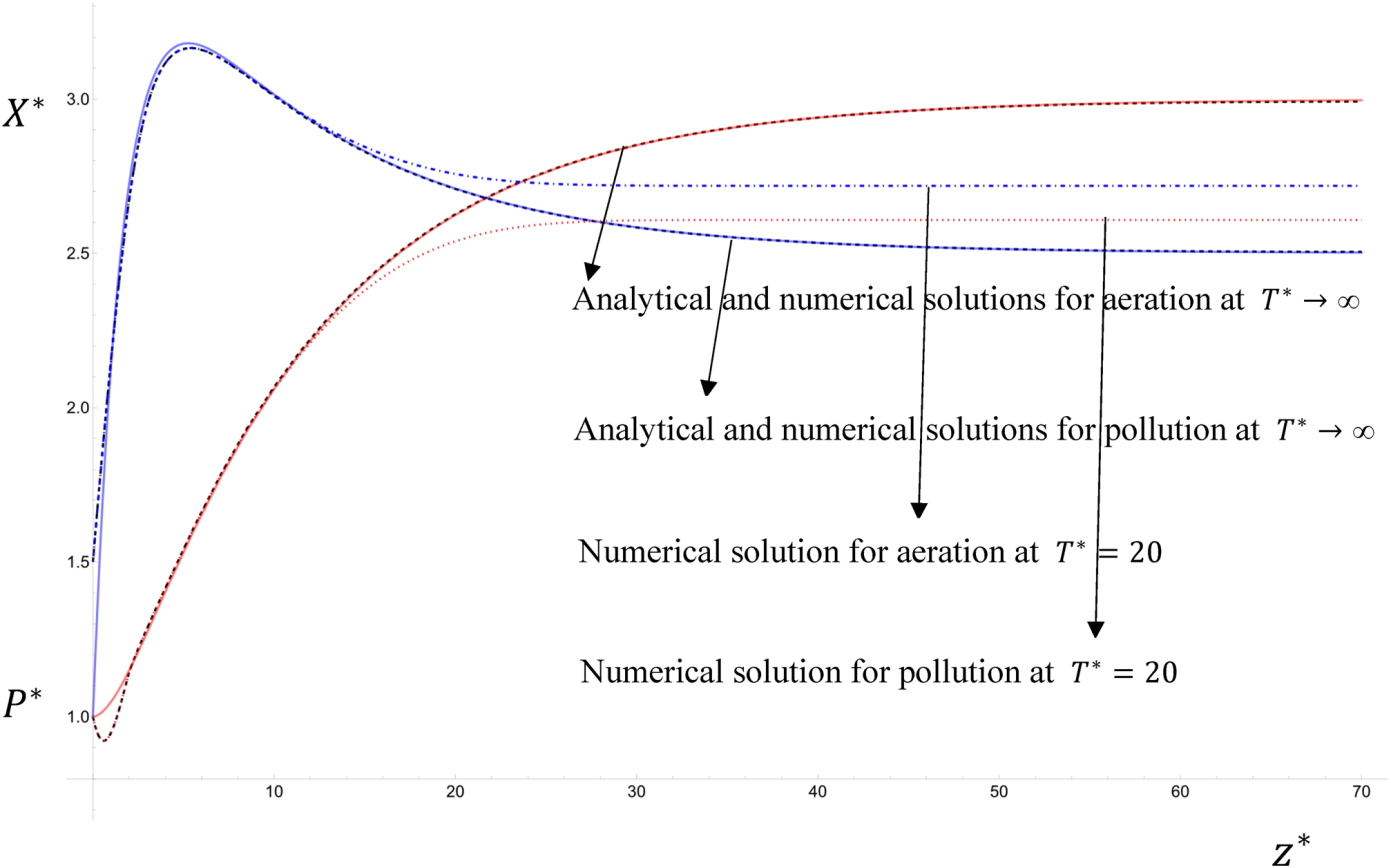
The comparison between the steady state analytical solutions given by Eqs. (28, 29) and the steady state of the numerical solution of Eqs. (30, 31).

Figure 4 shows a comparison between the steady state solution given by Eqs. (28, 29) and the steady state numerical solution of Eqs. (30, 37) (i.e. when the change with time ${T}^{*}$ is negligible, which is at ${T}^{*}\cong 60$ for the following parameter values) and an unsteady state numerical solution (for ${T}^{*}=20$), for the parameter values: ${K}_{1}^{*}=1.1$; ${K}_{2}^{*}=0.5$; ${S}^{*}=$ ${{\alpha }_{1}}^{*}=2$; ${{\eta }_{1}}^{*}={{ \eta }_{2}}^{*}=0.98$; ${\omega }^{*}=2\pi$; ${\lambda }^{*}=0.6$; ${R}_{0}=1$; ${P}_{0}^{*}=0.1$; ${{P}_{1}}^{*}=0$; ${P}_{3}^{*}=0.5$; ${X}_{0}^{*}=6$; ${{X}_{1}}^{*}=0$; ${X}_{3}^{*}=0.5$; ${k}^{*}=0$; ${U}^{*}={Pe}_{{P}^{*}}={Pe}_{{X}^{*}}=1$; ${\upmu }^{*}=0.3$; $0\le {z}^{*}\le 70$. A very good agreement is found between the steady-state solutions. It is seen that as ${T}^{*}$ increases in the numerical solution of the unsteady case, the numerical solution approaches the analytical solution in both pollution and aeration. This implies that the numerical solution can be used in both unsteady and steady cases. Thus, the tedious complications of the analytical solution are avoidable.

**Figure 5:**
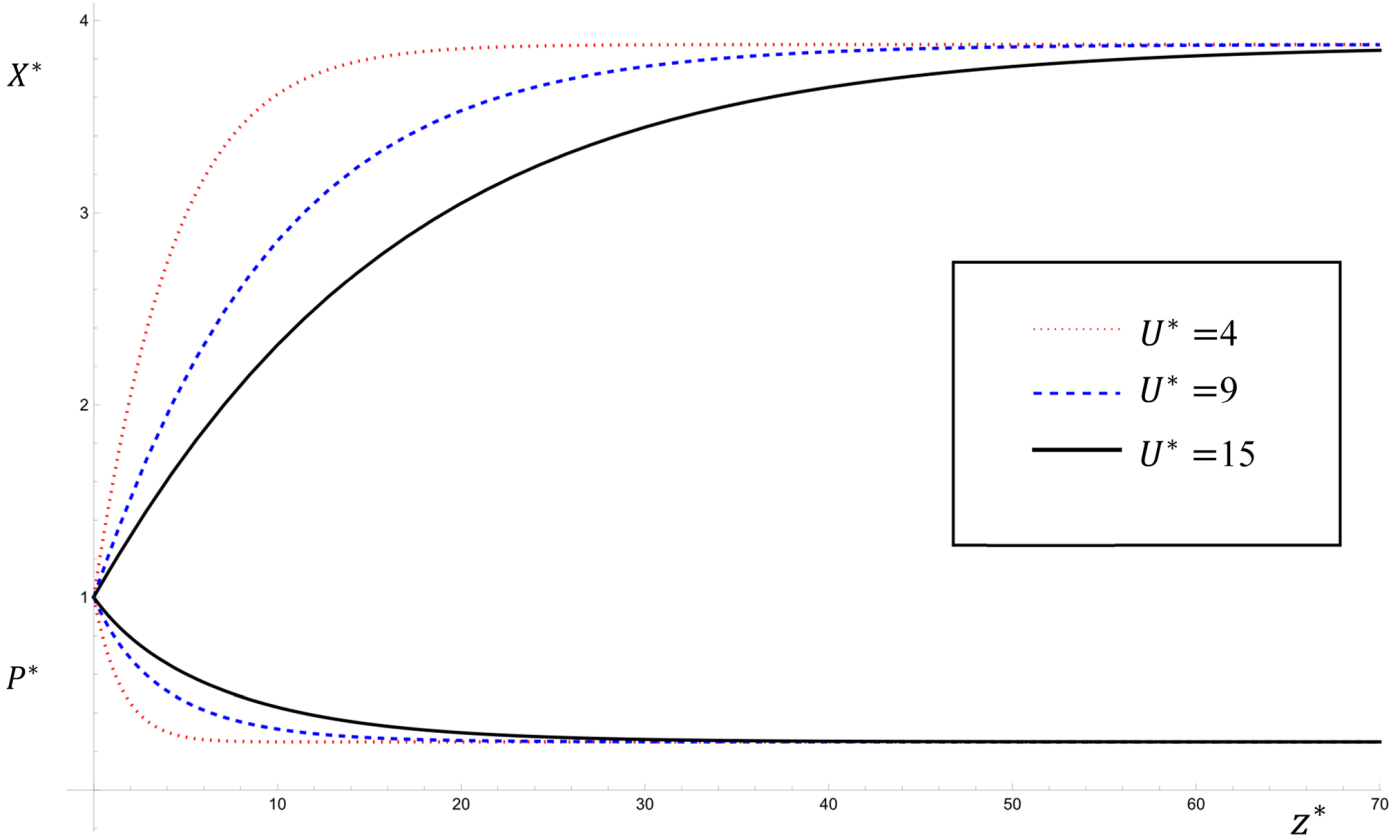
The variations of ${P}^{*}$ and ${X}^{*}$ with ${U}^{*}$ for the values ${U}^{*}=4, 9,15$ for the steady state analytical solutions given by Eqs. (28, 29).

Since the flux of water Q = *A* ${U}^{*}$(the volume of water crossing A per unit time), the flux Q increases as ${U}^{*}$ increases which directly leads to an increase in the value of ${P}^{*}$ while the value of ${X}^{*}$ decreases. Figure 5 shows the variations of ${P}^{*}$ and ${X}^{*}$ with ${U}^{*}$ for ${U}^{*}=4, 9, 15$ for the steady state solution given by Eqs. (28, 29) for the parameter values: $Pe=6$; ${K}_{1}^{*}=3$; ${\lambda }^{*}=0.6$; ${\upmu }^{*}=0.5$; ${K}_{2}^{*}=0.5$; ${S}^{*}=$ ${{\alpha }_{1}}^{*}=2$; $0\le {z}^{*}\le 70$. It is clear that near the source (${z}^{*}=0$), as ${U}^{*}$ increases the value of ${P}^{*}$ increases and the value of ${X}^{*}$ decreases. This result agrees with that obtained by^11,13^.

**Figure 6:**
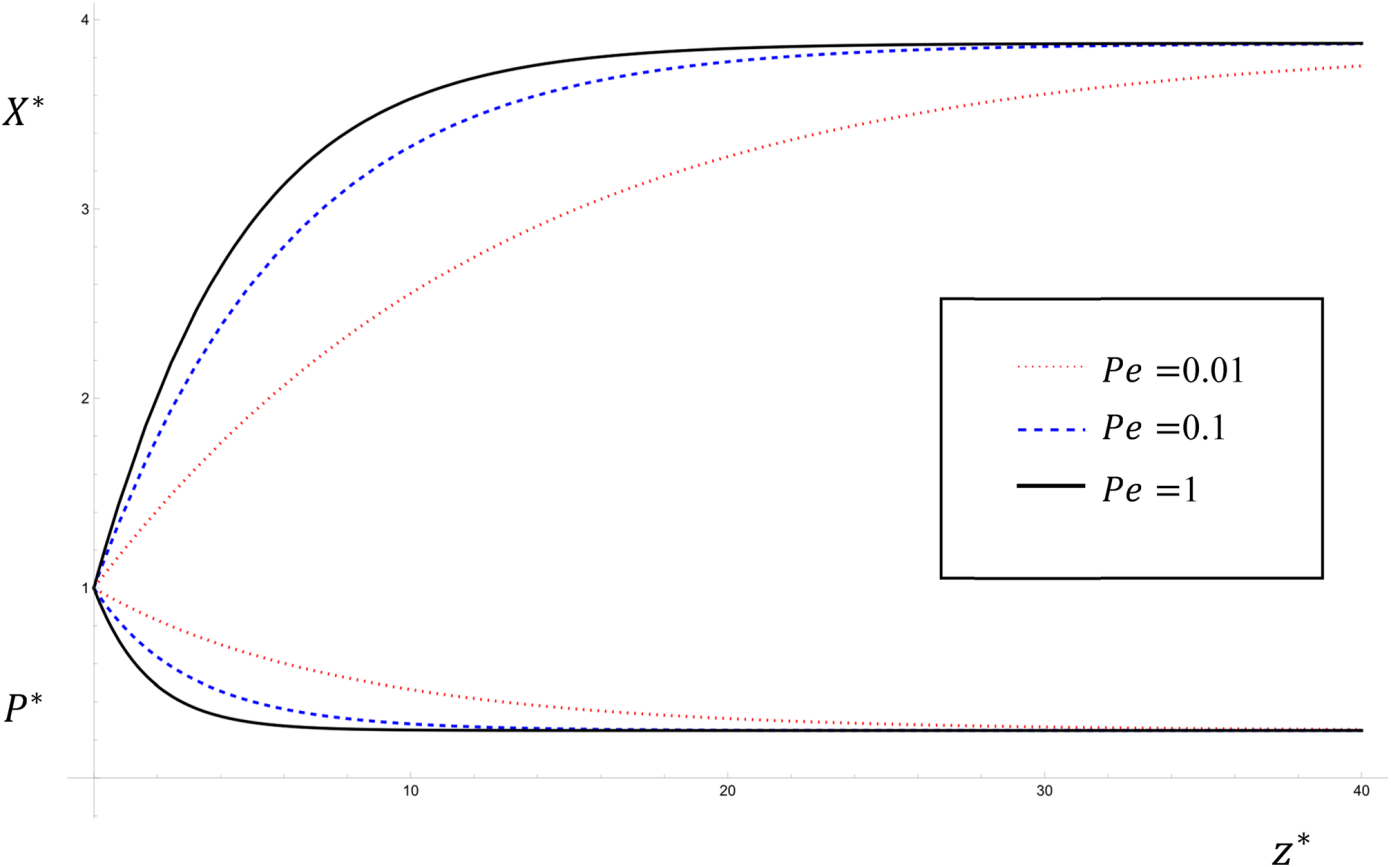
The variations of ${P}^{*}$ and ${X}^{*}$ with Peclet number for $Pe=0.01, 0.1, 1$ for the steady state analytical solutions given by Eqs. (28, 29).

It is known that the Peclet number represents the ratio between the advective transport rate and the diffusive transport rate. Consequently, while the value of the Peclet number remains less than one, the increase in the Peclet number leads to a decrease in the effect of diffusion and thus a decrease in pollutant concentration values. Figure 6 shows the variations of ${P}^{*}$ and ${X}^{*}$ with Peclet number for $Pe=\mathrm{0.01,0.1}, 1$ in the steady state solution given by Eqs. (28, 29) for the parameter values ${U}^{*}=4$; ${K}_{1}^{*}=3$; ${\lambda }^{*}=0.6$; ${\upmu }^{*}=0.5$; ${K}_{2}^{*}=0.5$; ${S}^{*}=$ ${{\alpha }_{1}}^{*}=2$; $0\le {z}^{*}\le 40$. It is seen that, near the source (${z}^{*}=0$), as the value of the Peclet number increases, the value of ${P}^{*}$ decreases and the value of ${X}^{*}$ increases. This result agrees with that obtained by^13,28^.

**Figure 7:**
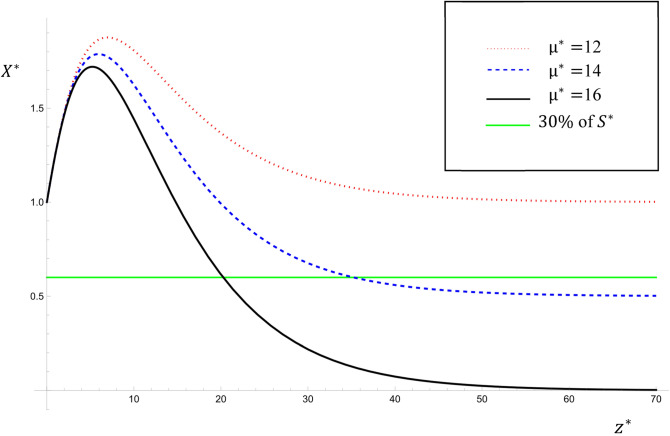
The variations of ${X}^{*}$ with ${\upmu }^{*}$ for ${\upmu }^{*}=12, 14, 16$ for the steady state analytical solutions given by Eqs. (28, 29).

Figure 7 shows the variations of ${X}^{*}$ with ${\upmu }^{*}$ for ${\upmu }^{*}=12, 14, 16$ for the steady state solution given by Eqs. (28 and 29) for the parameter values: $Pe=6$; ${K}_{1}^{*}=3$; ${\lambda }^{*}=0.6$; ${U}^{*}=9$; ${K}_{2}^{*}=0.5$; ${S}^{*}=$ ${{\alpha }_{1}}^{*}=2$; $0\le {z}^{*}\le 70$. As expected, as ${\upmu }^{*}$ increases the values of ${X}^{*}$ decrease along the river. This result agrees with that obtained by^4,7^. Since the fish will survive when ${X}^{*}>30\% {S}^{*}$, it is obvious that this restricts the value of ${\upmu }^{*}$ for a fixed interval of the river at a given time ${T}^{*}$. From Fig. 7, it is clear that for the given parameters, the region at which fish survive depends on the value of ${\upmu }^{*}$ as follows: for ${\upmu }^{*}$ = 16, the fish can survive only in the region ${z}^{*}\le 20$, for ${\upmu }^{*}$ = 14, the fish can survive only in the region ${z}^{*}\le 35$ and for ${\upmu }^{*}$ = 12, the fish can survive at any region along the river. Thus, the suitable interval along the river and the time for fishing can be determined with high accuracy. The importance of this figure is that it graphically explains the actual procedure, which is directly related to real-life situations requiring predictive tools to help with decision-making. The steps for determining the ideal place and time for placing fish farms are suggested as follows:

1. Taking measurements and calculating all the constant parameters of the model, such as (${Pe}_{{P}^{*}}$, ${S}^{*}$, ${{\alpha }_{1}}^{*}$,…).
2. Getting solutions and graphing a similar figure for an arbitrary value of ${\upmu }^{*}$.
3. Varying the values of ${\upmu }^{*}$ until the value of ${X}^{*}$ is sufficiently above the green (solid horizontal) line, which guarantees the survival of the fish.

The value of ${\upmu }^{*}$ obtained in this way is the maximum pollutant concentration that is allowed to be injected along the river for fish survival at a given time. Furthermore, for a fixed interval along the river, we see the change in ${X}^{*}$ (DO concentration) with respect to the different values of ${\upmu }^{*}$ which is essential in determining the ideal place for placing fish farms. It is seen that the procedure and the model are neither economically expensive nor have any environmental risk, and due to their simplicity, they could be put into practical experiments.

Table 1 presents the analytical solution in the steady-state case taken as a reference and shows a comparison between different step sizes in the finite difference method. We see from the table that $0.002$ is the most suitable value for Δ${T}^{*}$. Both Δ${z}^{*}=0.5$ and Δ${z}^{*}=1$ give acceptable results relative to the analytical solution.

**Table 1:** A comparison between the steady-state solutions using the finite difference method, NDSolve via Mathematica, and the analytical solution.

	The analytical solution given by Eqs. (28 and 29)	NDSolve via Mathematica software	Finite difference scheme at $\Delta T^{*} = 0.002\;and\;\Delta z^{*} = 1$	Finite difference scheme at $\Delta T^{*} = 0.002\;and\;\Delta z^{*} = 0.5$	Finite difference scheme at $\Delta T^{*} = 0.002\;and\;\Delta z^{*} = 2$	Finite difference scheme at $\Delta T^{*} = 0.001\;and\;\Delta z^{*} = 1$	Finite difference scheme at $\Delta T^{*} = 0.01\;and\;\Delta z^{*} = 1$
${P}^{*}\left(2\right)$	$1.1494533$	$1.1867367$	$1.1584454$	$1.1518907$	$1.1819467$	$1.1584436$	$1.1584599$
${P}^{*}\left(12\right)$	$2.2178496$	$2.2328495$	$2.222876$	$2.2191471$	$2.2356381$	$2.2228759$	$2.2228759$
${P}^{*}\left(26\right)$	$2.7830064$	$2.7872069$	$2.7840617$	$2.78327967$	$2.7866212$	$2.7840617$	$2.7840618$
${P}^{*}\left(28\right)$	$2.8193336$	$2.8228278$	$2.820169995$	$2.8195490$	$2.8221835$	$2.820169986$	$2.8201701$
${P}^{*}\left(46\right)$	$2.9652664$	$2.9655902$	$2.9650539$	$2.9649849$	$2.9652446$	$2.9650533$	$2.9650583$
${X}^{*}\left(2\right)$	$2.7250323$	$2.6051910$	$2.6902307$	$2.7154925$	$2.6059493$	$2.6902337$	$2.6902063$
${X}^{*}\left(12\right)$	$2.9323526$	$2.922129995$	$2.9290927$	$2.9315182$	$2.9204128$	$2.9290927$	$2.9290927$
${X}^{*}\left(26\right)$	$2.6205515$	$2.6182158$	$2.6199649$	$2.6203996$	$2.6185417$	$2.6199649$	$2.6199649$
${X}^{*}\left(28\right)$	$2.6003701$	$2.59842798$	$2.5999053$	$2.6002504$	$2.5987862$	$2.5999053$	$2.5999053$
${X}^{*}\left( 46\right)$	$2.5192965$	$2.5191166$	$2.5194145$	$2.5194528$	$2.5193085$	$2.5194148$	$2.5194121$

**Figure 8:**
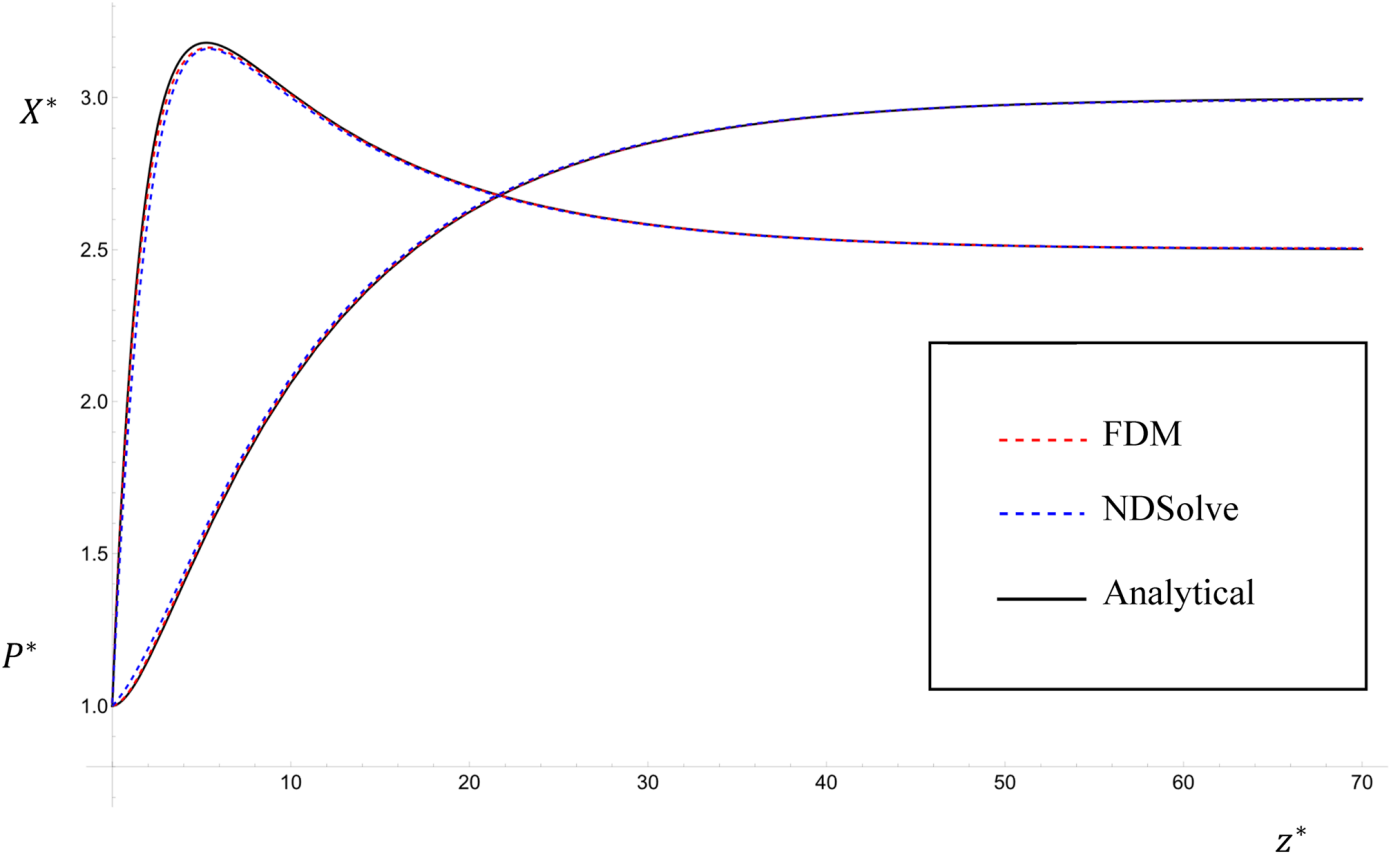
A comparison between the steady-state solutions using the finite difference method (FDM), NDSolve via Mathematica, and the analytical solution given by Eqs. (28, 29).

Furthermore, Table 1 and Fig. 8 illustrate a comparison between the steady-state analytical solution and the finite difference method and NDSolve via Mathematica. From the table, it is clear that the finite difference scheme is more accurate than NDSolve in this case. In conclusion, a good agreement was found between the three solutions, which is clear in Fig. 8.

It should be noted that when the amplitudes of sine or cosine functions (i.e., ${P}_{3}^{*}$ or ${X}_{3}^{*}$) are large in relation to the other parameters of the model, it results in a negative value of the concentrations, which deviates from the real-life meaning.

A final comment on the comparison between case studies one and two: While case study two generalizes previous models, case study one presents a preferable model to get useful information due to the presence of ${\upmu }^{*}$ representing the amount of injected pollutants along the river. Thus, obtaining the maximum threshold which acts as guidance towards making necessary decisions.

## Conclusion

- A generalized pollution-aeration model was established by using time as well as spatial-dependent parameters. Also, general boundary constraints were considered in the two case studies, and special cases of the proposed model agree with its predecessors, making it advantageous to be used in global cases.
- The developed model was solved analytically in the steady state case and numerically in the unsteady state case using the finite difference method. A comparison is made between the analytical solution in the steady state case and the numerical solution in its steady state. Also, Mathematica software was used via NDSolve for a comparison with the finite difference method as well as the analytical solution in the steady-state case. In conclusion, a very good agreement is found.
- Case study one, i.e., when considering the injected pollutants along the river, is beneficial in decision-making regarding the regulation of industrial and agricultural practices and, if needed, restricting them. Since tons of fish die every year in the Rosetta Branch of the Nile River in Egypt and other rivers suffer from the same problem as well, this study gives a suitable solution to this problem. The suitable time for fishing as well as the location of fish farms are determined with high accuracy by following a simple step-by-step procedure.

## Limitations and recommendations

This work is limited by the usage of a conditionally-stable numerical method, namely, the finite difference method. The analysis carried out revealed that for the given parameter values, the results are satisfactory. However, using an unconditionally-stable numerical method such as the Crank-Nicolson method for example, will allow for even wider parameter values. Hence, analyzing the general aeration model using different numerical methods could be a fruitful area of research. Also, considering different boundary conditions such as pulse-type and mixed-type conditions is recommended.
